# Outcome after CPR: when we cannot save lives, we can save organs

**DOI:** 10.1186/cc14520

**Published:** 2015-03-16

**Authors:** C Caestecker, J Froyman, P Lormans, W Stockman

**Affiliations:** 1 AZ Delta, Roeselare, Belgium

## Introduction

Patients resuscitated after cardiac arrest (CA) who suffer bad neurologic outcome or become brain dead might become organ donors (OD) when well managed and identified. We report on the cohort of patients resuscitated after in-hospital or out-of-hospital CA becoming OD in a tertiary community hospital with an intensive donor identification program.

## Methods

Data from our 28-bed mixed medical/surgical adult ICU in a 900-bed tertiary hospital were analyzed from 2010 to 2014.

## Results

Our ICU admitted 2,320 patients/year. Overall ICU mortality in this period was 7.4%. A summary of the results is presented in Table 1. Of the 219 patients admitted after CA, 21 (10%) became OD. This resulted in 55 successfully transplanted organs (28 kidneys, 17 livers, seven lung pairs, three hearts). Of note, good outcome (CPC 1 and 2) was achieved in 55 patients (25%).

**Table 1:** 

Year	Organ donors	Donors after CA	DCD/DBD	ICU admission after CA
2010	11	3	0/3	40
2011	9	5	3/2	41
2012	18	4	3/1	47
2013	17	5	4/1	53
2014	13	4	1/3	38
Total	68	21 (31%)	11/10	219

## Conclusion

Ten percent of patients resuscitated after CA and admitted to the ICU become OD, consisting of up to 31% of the total number of OD in our center. Patients resuscitated after CA who suffer severe irreversible brain damage or are brain dead can thus substantially expand the donor pool. This justifies extensive resuscitation efforts, if not to save lives, then to save organs. This might be reassuring for families, staff and the community.

